# Supplementary figures and images for: Comparing online versus laboratory measures of speech perception in older children and adolescents
Source: PLoS One. 2024 Feb 7;19(2):e0297530. doi: 10.1371/journal.pone.0297530 (PMC10849252; doi:10.1371/journal.pone.0297530)

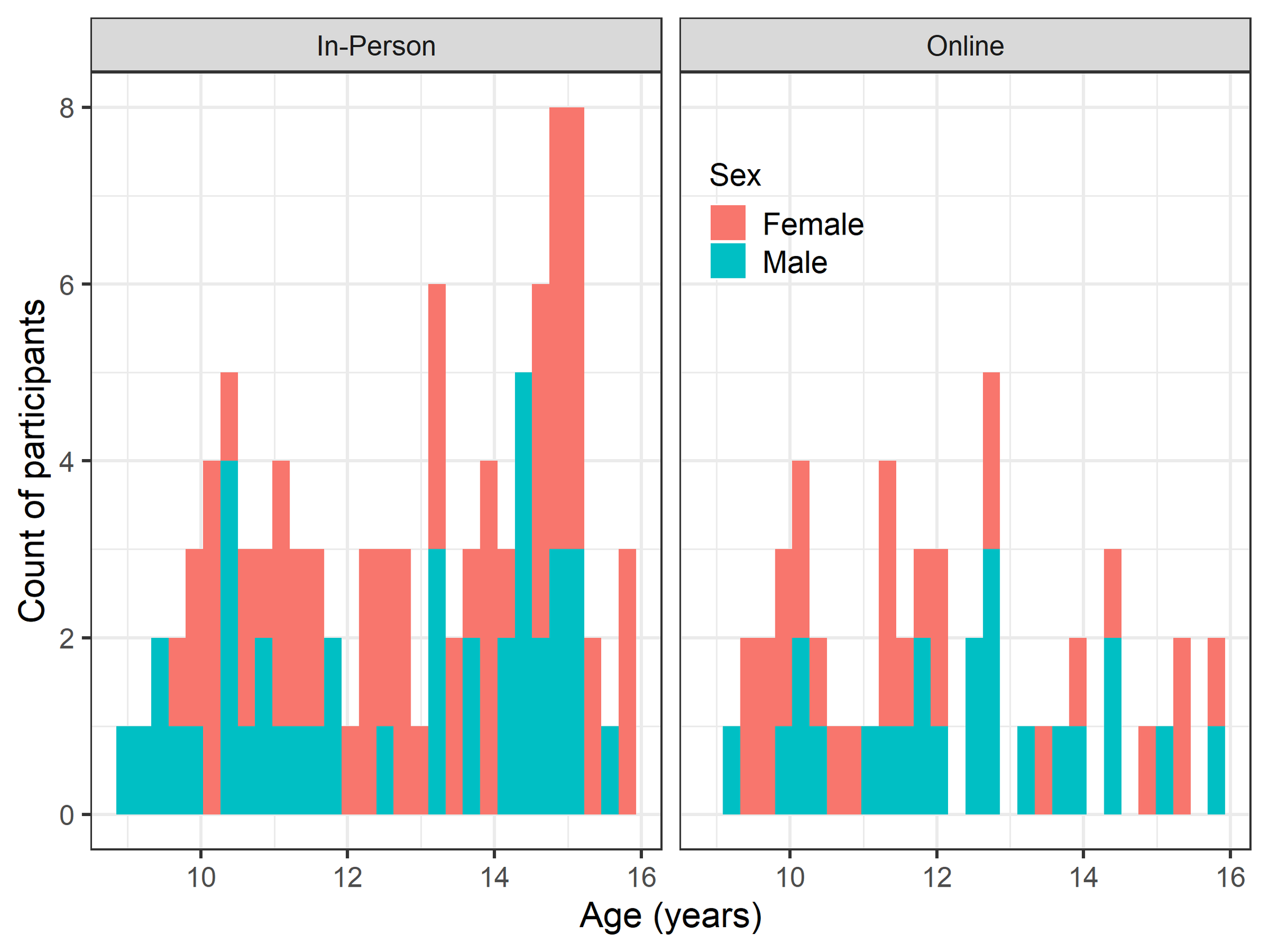

Supplement: S1 Fig — (TIF) [file pone.0297530.s001.tif]
