# Supplementary material for: Comparing online versus laboratory measures of speech perception in older children and adolescents
Source: PLoS One. 2024 Feb 7;19(2):e0297530. doi: 10.1371/journal.pone.0297530 (PMC10849252; doi:10.1371/journal.pone.0297530)
Supplement: S1 Table — (DOCX) [file pone.0297530.s002.docx]

**S1 Table: Individual-level characteristics of included participants in the in-person setting. GFTA-3=Goldman-Fristoe Test of Articulation-3; CELF-5=Clinical Evaluation of Language Fundamentals-5 Screening Test; NYU = New York University; MSU = Montclair State University; SU = Syracuse University.**

| **Study ID** | **Age (years)** | **Sex** | **GFTA (standard score)** | **CELF-5 Screener (Total Raw Score)** | **Site** |
| --- | --- | --- | --- | --- | --- |
| 1008 | 12.76 | Female | 104 | 24 | NYU |
| 1009 | 9.88 | Male | 108 | 22 | NYU |
| 1010 | 11.87 | Male | 105 | 25 | NYU |
| 1012 | 14.48 | Male | 103 | 27 | NYU |
| 1013 | 14.77 | Female | 103 | 30 | NYU |
| 1015 | 9.43 | Male | 108 | 27 | NYU |
| 1016 | 14.82 | Male | 103 | 26 | NYU |
| 1017 | 10.54 | Female | 105 | 25 | NYU |
| 1018 | 14.66 | Female | 103 | 28 | NYU |
| 1019 | 11.38 | Male | 105 | 22 | NYU |
| 1021 | 10.92 | Female | 105 | 20 | NYU |
| 1022 | 14.81 | Female | 103 | 28 | NYU |
| 1023 | 13.68 | Male | 103 | 24 | NYU |
| 1025 | 10.04 | Female | 105 | 21 | NYU |
| 1026 | 11.1 | Female | 104 | 23 | NYU |
| 1029 | 12.4 | Male | 99 | 23 | NYU |
| 1030 | 12.4 | Female | 104 | 25 | NYU |
| 1031 | 13.11 | Male | 104 | 25 | NYU |
| 1032 | 14.55 | Female | 103 | 27 | NYU |
| 1034 | 9.45 | Male | 106 | 16 | NYU |
| 1037 | 11.63 | Female | 104 | 29 | NYU |
| 1038 | 11.38 | Female | 104 | 30 | NYU |
| 1039 | 11.91 | Male | 105 | 28 | NYU |
| 1040 | 12.18 | Female | 104 | 25 | NYU |
| 1041 | 15.76 | Female | 103 | 28 | NYU |
| 1043 | 15.05 | Male | 103 | 24 | NYU |
| 1046 | 15.21 | Female | 103 | 28 | NYU |
| 1047 | 9.04 | Male | 101 | 14 | NYU |
| 1048 | 10.11 | Female | 105 | 28 | NYU |
| 1049 | 14.34 | Male | 103 | 26 | NYU |
| 1050 | 10.93 | Male | 106 | 28 | NYU |
| 1051 | 10.64 | Female | 105 | 28 | NYU |
| 1052 | 14.34 | Male | 103 | 19 | NYU |
| 1054 | 10.59 | Male | 107 | 25 | NYU |
| 1055 | 9.71 | Female | 106 | 19 | NYU |
| 1056 | 15.71 | Female | 103 | 29 | NYU |
| 1057 | 13.92 | Female | 103 | 31 | NYU |
| 1058 | 15.16 | Male | 103 | 27 | NYU |
| 3000 | 10.01 | Female | 108 | 18 | MSU |
| 3001 | 13.69 | Female | 107 | 21 | MSU |
| 3002 | 10.35 | Male | 98 | 21 | MSU |
| 3003 | 13.22 | Female | 103 | 25 | MSU |
| 3004 | 12.76 | Female | 107 | 26 | MSU |
| 3005 | 12.47 | Female | 104 | 25 | MSU |
| 3006 | 15.2 | Male | 102 | 23 | MSU |
| 3009 | 12.26 | Female | 107 | 25 | MSU |
| 3010 | 14.06 | Male | 108 | 25 | MSU |
| 3011 | 14.89 | Female | 106 | 25 | MSU |
| 3012 | 12.34 | Female | 107 | 25 | MSU |
| 3013 | 10.44 | Male | 110 | 23 | MSU |
| 3014 | 13.79 | Male | 104 | 28 | MSU |
| 3015 | 14.57 | Male | 103 | 26 | MSU |
| 3016 | 14.83 | Female | 102 | 26 | MSU |
| 3018 | 13.39 | Female | 103 | 24 | MSU |
| 3020 | 14.62 | Female | 103 | 28 | MSU |
| 3021 | 14.62 | Male | 103 | 26 | MSU |
| 3024 | 14.28 | Female | 103 | 24 | MSU |
| 3025 | 10.03 | Female | 105 | 23 | MSU |
| 3026 | 14.07 | Male | 103 | 25 | MSU |
| 3027 | 15.43 | Female | 103 | 29 | MSU |
| 3028 | 11.35 | Female | 104 | 19 | MSU |
| 3029 | 14.65 | Female | 103 | 24 | MSU |
| 3030 | 13.19 | Male | 98 | 19 | MSU |
| 3031 | 15.52 | Male | 103 | 25 | MSU |
| 3036 | 11.5 | Male | 105 | 23 | MSU |
| 3043 | 15.11 | Female | 103 | 24 | MSU |
| 3044 | 15.75 | Female | 103 | 28 | MSU |
| 3045 | 13.3 | Male | 104 | 22 | MSU |
| 3046 | 10.42 | Male | 100 | 24 | MSU |
| 3047 | 13.14 | Female | 107 | 20 | MSU |
| 3048 | 10.35 | Female | 105 | 20 | MSU |
| 3049 | 13.32 | Female | 103 | 26 | MSU |
| 3051 | 15.06 | Female | 103 | 28 | MSU |
| 3055 | 13.86 | Female | 103 | 21 | MSU |
| 3056 | 10.03 | Female | 105 | 24 | MSU |
| 3057 | 12.8 | Female | 104 | 22 | MSU |
| 3059 | 11.6 | Female | 104 | 25 | MSU |
| 3061 | 15.06 | Female | 103 | 27 | MSU |
| 3063 | 10.82 | Male | 106 | 15 | MSU |
| 3064 | 10.99 | Female | 105 | 26 | MSU |
| 3066 | 9.89 | Female | 106 | 23 | MSU |
| 3067 | 14.9 | Male | 103 | 25 | MSU |
| 3068 | 13.47 | Female | 103 | 19 | MSU |
| 3069 | 15.43 | Female | 103 | 22 | MSU |
| 3070 | 14.46 | Male | 103 | 26 | MSU |
| 6001 | 13.91 | Female | 103 | 23 | SU |
| 6002 | 12.12 | Female | 104 | 17 | SU |
| 6003 | 13.91 | Female | 103 | 26 | SU |
| 6004 | 12.94 | Female | 104 | 25 | SU |
| 6005 | 15.14 | Female | 103 | 26 | SU |
| 6006 | 11.01 | Male | 105 | 26 | SU |
| 6007 | 14.3 | Male | 103 | 24 | SU |
| 6008 | 11.14 | Female | 104 | 24 | SU |
| 6009 | 14.75 | Male | 103 | 28 | SU |
| 6010 | 9.27 | Male | 99 | 16 | SU |
| 6013 | 14.95 | Female | 103 | 25 | SU |
| 6014 | 10.48 | Male | 100 | 20 | SU |
| 6015 | 9.7 | Male | 108 | 22 | SU |
