# Supplementary material for: Comparing online versus laboratory measures of speech perception in older children and adolescents
Source: PLoS One. 2024 Feb 7;19(2):e0297530. doi: 10.1371/journal.pone.0297530 (PMC10849252; doi:10.1371/journal.pone.0297530)
Supplement: S2 Table — (DOCX) [file pone.0297530.s003.docx]

**S2 Table: Individual-level characteristics of included participants in the online modality. GFTA-3=Goldman-Fristoe Test of Articulation-3; CELF-5=Clinical Evaluation of Language Fundamentals-5; FS=Formulated Sentences subtest on CELF-5; RS=Recalling Sentences subtest on CELF-5; MSU=Montclair State University; SU=Syracuse University.**

| **Study ID** | **Age (years)** | **Sex** | **GFTA-3 (standard score)** | **CELF-5 FS (scaled score)** | **CELF-5 RS (scaled score)** | **Site** |
| --- | --- | --- | --- | --- | --- | --- |
| 8000 | 10.20 | Female | 105 | 8 | 7 | MSU |
| 8001 | 12.01 | Female | 104 | 10 | 8 | MSU |
| 8002 | 14.80 | Female | 103 | 9 | 9 | MSU |
| 8003 | 12.67 | Male | 105 | 10 | 11 | MSU |
| 8004 | 9.10 | Female | 106 | 13 | 16 | MSU |
| 8005 | 11.36 | Female | 104 | 12 | 9 | MSU |
| 8006 | 14.02 | Male | 103 | 12 | 17 | MSU |
| 8007 | 11.43 | Female | 104 | 13 | 10 | MSU |
| 8008 | 9.64 | Female | 106 | 15 | 13 | MSU |
| 8009 | 9.38 | Female | 106 | 12 | 11 | MSU |
| 8011 | 13.92 | Female | 103 | 10 | 14 | MSU |
| 8012 | 12.13 | Male | 105 | 10 | 12 | MSU |
| 8013 | 9.70 | Female | 106 | 13 | 15 | SU |
| 8014 | 12.65 | Male | 105 | 14 | 14 | MSU |
| 8015 | 12.73 | Male | 105 | 12 | 16 | MSU |
| 8016 | 9.53 | Female | 106 | 15 | 15 | MSU |
| 8017 | 15.88 | Male | 103 | 15 | 10 | MSU |
| 8018 | 14.33 | Female | 103 | 15 | 12 | MSU |
| 8019 | 15.21 | Male | 103 | 9 | 8 | MSU |
| 8020 | 10.34 | Male | 107 | 10 | 8 | MSU |
| 8021 | 13.37 | Female | 103 | 13 | 10 | MSU |
| 8022 | 15.23 | Female | 103 | 9 | 8 | MSU |
| 8024 | 10.88 | Female | 105 | 11 | 13 | MSU |
| 8025 | 11.72 | Female | 104 | 15 | 11 | MSU |
| 8026 | 13.58 | Male | 104 | 9 | 11 | MSU |
| 8027 | 10.12 | Female | 105 | 10 | 9 | MSU |
| 8028 | 15.27 | Female | 103 | 9 | 9 | MSU |
| 8029 | 10.12 | Male | 107 | 13 | 10 | MSU |
| 8031 | 10.63 | Female | 105 | 13 | 15 | SU |
| 8032 | 11.63 | Female | 104 | 11 | 9 | MSU |
| 8033 | 9.94 | Female | 106 | 15 | 12 | SU |
| 8034 | 11.17 | Male | 105 | 11 | 14 | MSU |
| 8035 | 11.92 | Female | 104 | 12 | 14 | SU |
| 8036 | 15.89 | Female | 103 | 11 | 14 | SU |
| 8037 | 11.84 | Male | 105 | 10 | 12 | MSU |
| 8038 | 14.42 | Male | 103 | 14 | 10 | MSU |
| 8039 | 14.45 | Male | 103 | 7 | 8 | MSU |
| 8040 | 13.31 | Male | 104 | 15 | 11 | SU |
| 8041 | 12.59 | Male | 105 | 14 | 11 | SU |
| 8042 | 11.39 | Female | 104 | 9 | 10 | MSU |
| 8044 | 11.83 | Male | 105 | 7 | 10 | MSU |
| 8049 | 12.64 | Male | 105 | 18 | 12 | MSU |
| 8050 | 10.33 | Female | 105 | 17 | 14 | MSU |
| 8052 | 11.58 | Male | 105 | 15 | 11 | MSU |
| 8053 | 12.55 | Male | 105 | 15 | 14 | SU |
| 8054 | 11.37 | Male | 105 | 12 | 15 | MSU |
| 8056 | 10.11 | Male | 107 | 13 | 10 | MSU |
| 8057 | 10.01 | Male | 107 | 9 | 9 | MSU |
| 8058 | 10.01 | Female | 105 | 9 | 10 | MSU |
| 8059 | 12.86 | Female | 104 | 12 | 15 | MSU |
