# Supplementary material for: Comparing online versus laboratory measures of speech perception in older children and adolescents
Source: PLoS One. 2024 Feb 7;19(2):e0297530. doi: 10.1371/journal.pone.0297530 (PMC10849252; doi:10.1371/journal.pone.0297530)
Supplement: S3 Table — Includes measures of goodness of model fit, model diagnostics, and estimated marginal means. (DOCX) [file pone.0297530.s004.docx]

**S3 Table. Complete results of the linear model examining identification boundary width.**

***Original model*: The reference level for the variable of Modality was In-Person and the reference level for the variable of Sex was Female.**

**Model formula: identification = α + β1(Modality_Online_) + β2(Age) + β3(Sex_Male_) + β4(Modality_Online_ ×Age) + β5(Modality_Online_ × Sex_Male_) +** ε

| **Term** | **Estimate** | **Std. Error** | **Statistic** | **P-value** |
| --- | --- | --- | --- | --- |
| (Intercept) | 0.64 | 0.12 | 5.47 | **< 0.001** |
| Modality (Online) | 0.50 | 0.21 | 2.39 | **0.018** |
| Age | -0.04 | 0.05 | -0.99 | 0.325 |
| Sex (Male) | 0.41 | 0.18 | 2.31 | **0.023** |
| Modality (Online):Age | -0.17 | 0.08 | -2.12 | **0.036** |
| Modality (Online):Sex (Male) | -0.69 | 0.30 | -2.28 | **0.024** |

***Releveled model*: The reference level for the variable of Modality was Online and the reference level for the variable of Sex was Male.**

**Model formula: identification = α + β1(Modality_In-person_) + β2(Age) + β3(Sex_Female_) + β4(Modality_In-person_ ×Age) + β5(Modality_In-person_ × Sex_Female_) +** ε

| **Term** | **Estimate** | **Std. Error** | **Statistic** | **P-value** |
| --- | --- | --- | --- | --- |
|  |  |  |  |  |
| (Intercept) | 0.85 | 0.18 | 4.76 | **< 0.001** |
| Modality (In-person) | 0.20 | 0.23 | 0.88 | 0.38 |
| Age | -0.21 | 0.07 | -3.25 | **0.001** |
| Sex (Female) | 0.28 | 0.25 | 1.14 | 0.25 |
| Modality (In-person):Age | 0.17 | 0.08 | 2.12 | **0.036** |
| Modality (In-person):Sex (Female) | -0.69 | 0.30 | -2.28 | **0.024** |

**Goodness of fit for both models:**

Residual standard error: 0.86 on 140 degrees of freedom (2 observations deleted due to missingness)

Multiple R-squared: 0.15, Adjusted R-squared: 0.12

F-statistic: 4.86 on 5 and 140 DF, p-value: 0.0004

**Model Diagnostics:**


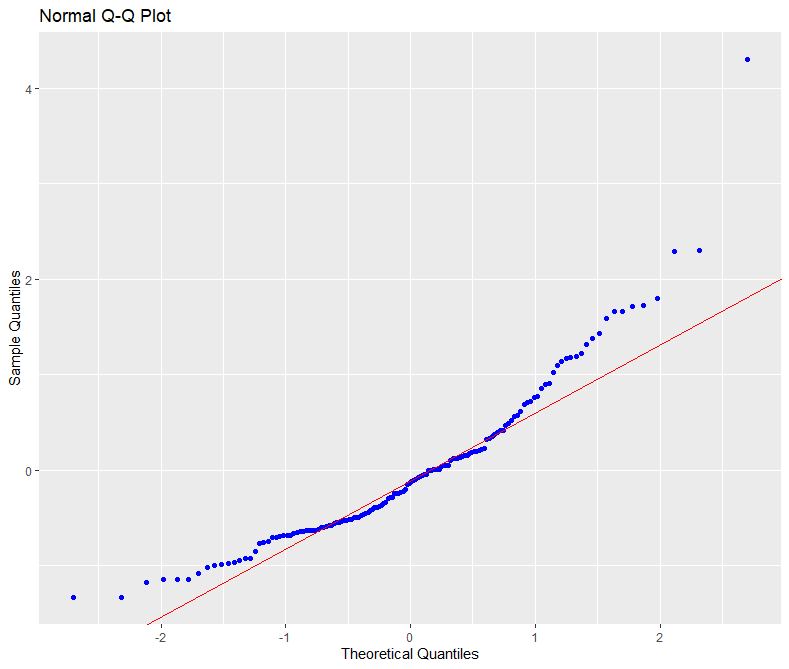

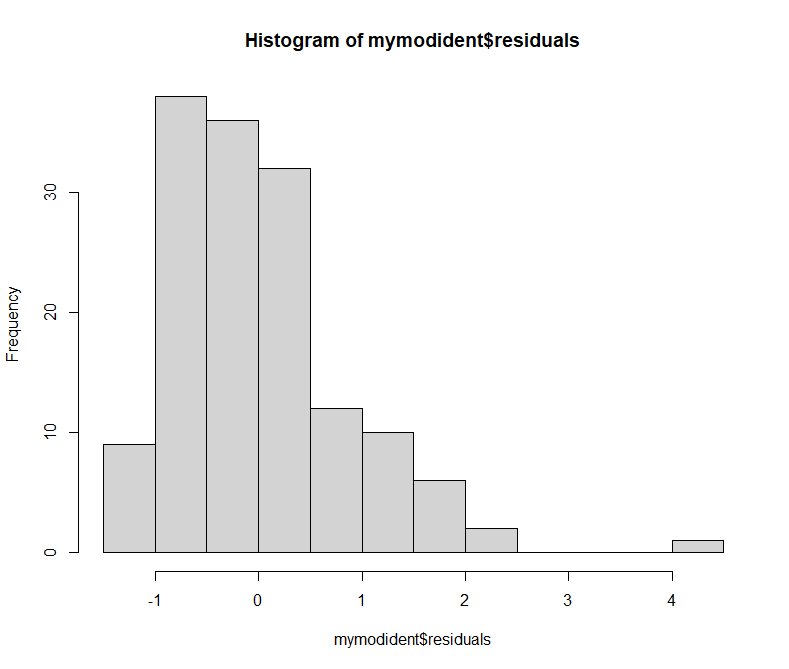


The null hypothesis of normality of residuals was rejected (Shapiro-Wilk W = .90, p < .001). However, current statistical thinking (Schmidt & Finan, 2018) suggests that this is not an issue when (a) the sample size is sufficiently large [greater than 10 observations per variable), (b) the focus is on interpreting coefficients rather than making predictions. Furthermore, this research suggests that methods to transform outcomes to satisfy the assumption of normality of residuals can bias model estimates and are not recommended.

Schmidt, A. F., & Finan, C. (2018). Linear regression and the normality assumption. *Journal of Clinical Epidemiology, 98*, 146-151.

**Estimated Marginal Means:**

**
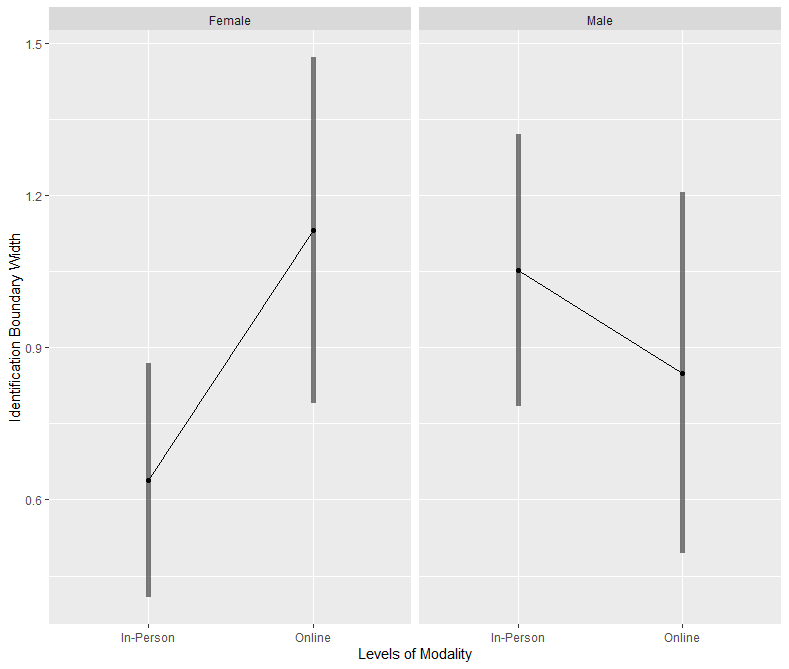
**
