# Supplementary material for: Comparing online versus laboratory measures of speech perception in older children and adolescents
Source: PLoS One. 2024 Feb 7;19(2):e0297530. doi: 10.1371/journal.pone.0297530 (PMC10849252; doi:10.1371/journal.pone.0297530)

**S4 Table. Complete results of the linear model examining category goodness percent correct.**

***Original model*: The reference level for the variable of Modality was In-Person and the reference level for the variable of Sex was Female.**

**Model formula: categorygoodness = α + β1(Modality_Online_) + β2(Age) + β3(Sex_Male_) + β4(Modality_Online_ ×Age) + β5(Modality_Online_ × Sex_Male_) +** ε

| **Term** | **Estimate** | **Std. Error** | **Statistic** | **P-value** |
| --- | --- | --- | --- | --- |
| (Intercept) | 85.19 | 1.24 | 68.43 | **< 0.001** |
| Modality (Online) | -2.38 | 2.24 | -1.06 | 0.29 |
| Age | 1.64 | 0.49 | 3.37 | **0.001** |
| Sex (Male) | -4.38 | 1.96 | -2.24 | **0.027** |
| Modality (Online):Age | -0.022 | 0.86 | -0.026 | 0.98 |
| Modality (Online):Sex (Male) | 7.03 | 3.30 | 2.13 | **0.035** |

***Releveled model*: The reference level for the variable of Modality was Online and the reference level for the variable of Sex was Male.**

**Model formula: categorygoodness = α + β1(Modality_In-person_) + β2(Age) + β3(Sex_Female_) + β4(Modality_In-person_ ×Age) + β5(Modality_In-person_ × Sex_Female_) +** ε

| **Term** | **Estimate** | **Std. Error** | **Statistic** | **P-value** |  |
| --- | --- | --- | --- | --- | --- |
|  |  |  |  |  |  |
| (Intercept) | 85.46 | 1.94 | 43.96 | **< 0.001** |  |
| Modality (In-person) | -4.65 | 2.46 | -1.89 | 0.061 |  |
| Age | 1.62 | 0.71 | 2.27 | **0.024** |  |
| Sex (Female) | -2.65 | 2.66 | -1.00 | 0.320 |  |
| Modality (In-person):Age | 0.02 | 0.86 | 0.03 | 0.979 |  |
| Modality (In-person):Sex (Female) | 7.03 | 3.30 | 2.13 | **0.035** |  |

**Goodness of fit for both models:**

Residual standard error: 9.3 on 139 degrees of freedom (3 observations deleted due to missingness)

Multiple R-squared: 0.15, Adjusted R-squared: 0.12

F-statistic: 5.02 on 5 and 139 DF, p-value: 0.0003

**Model Diagnostics:**


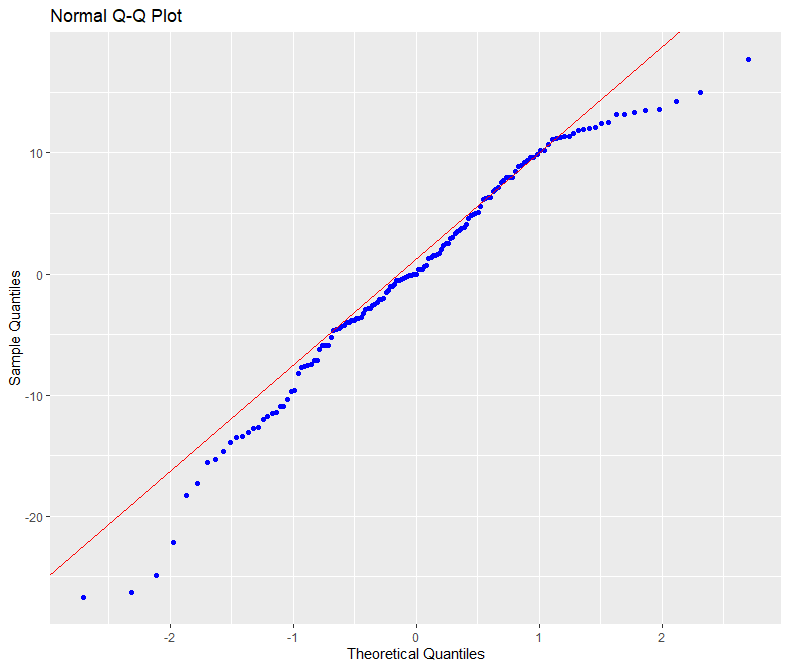

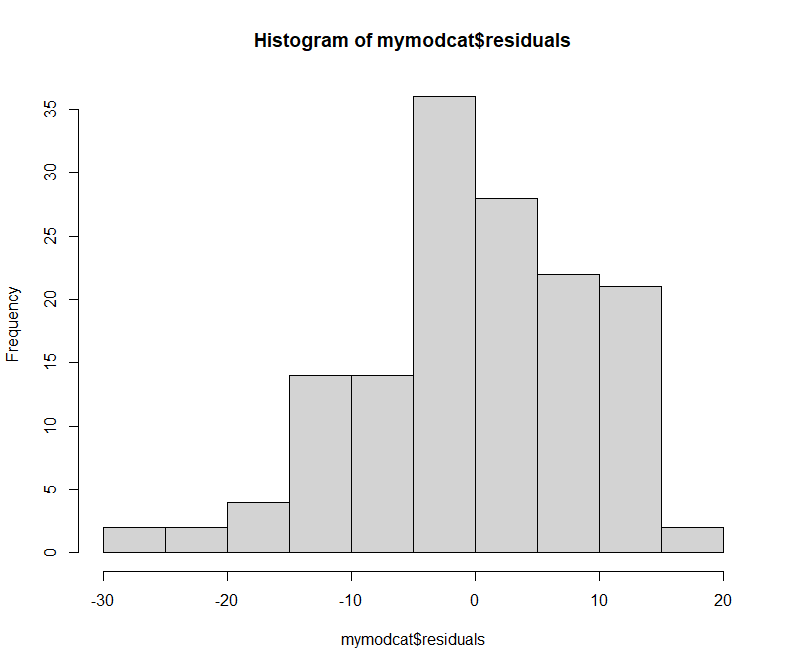


The null hypothesis of normality of residuals was rejected (Shapiro-Wilk W = .97, p = .005). However, we do not find the observed degree of non-normality concerning in light of the robust sample size; see note from Table S3 above.

**Estimated Marginal Means:**


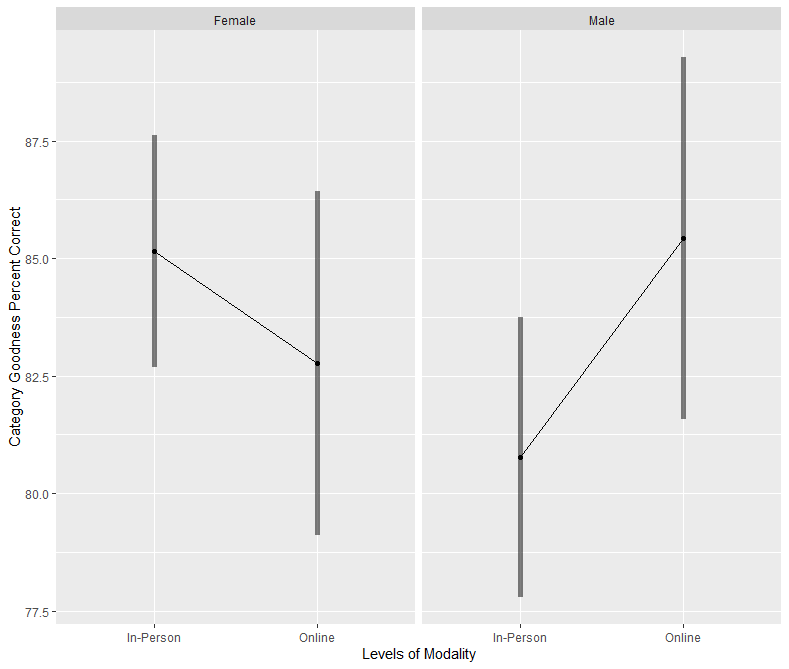

Supplement: S4 Table — Includes measures of goodness of model fit, model diagnostics, and estimated marginal means. (DOCX) [file pone.0297530.s005.docx]
