# Supplementary material for: Comparing online versus laboratory measures of speech perception in older children and adolescents
Source: PLoS One. 2024 Feb 7;19(2):e0297530. doi: 10.1371/journal.pone.0297530 (PMC10849252; doi:10.1371/journal.pone.0297530)
Supplement: Attachment — Submitted filename: Reviewer_Comments.docx [file pone.0297530.s006.docx]

**Summary**

In their manuscript entitled “*Comparing online versus laboratory measures of speech perception in older children and adolescents”*, the authors present data from an online and an in-person behavioural study (the in-person study is also partly described in a previous paper from some of the present authors) concerning speech perception in children and adolescents, suggesting that data collected online are no less valid than data collected in the laboratory.

Both in the online and the in-person study, they implemented a computerized identification task in which English speaking listeners had to report whether they heard the word “*rake*” or “*wake*” after listening to resynthesized auditory stimuli (previously recorded from one female speaker) derived from a continuum in which the first phoneme ranged from /ɹ/ to /w/ phoneme categories. Secondly, they implemented a Category Goodness Judgement task in which listeners heard several different words embedding the /ɹ/ phoneme recorded by multiple speakers with and without RSSD (residual speech sound disorder) and judged whether said words were phonetically realized in a correct way.

Results showed that the identification performance differed between the online and the in-person modalities, which authors linked to possible differences in noise level between the two, putatively influencing the percentage of participants with ceiling scores. No differences were reported for the goodness judgment. Additionally, identification performance and the goodness judgment performance correlated in the online task but not in the in-person task; the difference between the two correlation weights was not significant. Lastly, different interaction effects emerged between “Sex”, “Age” and “Modality (online vs in-person)” which authors attempted to link to different distributions of sex and age between the online and the in-person study.

This study shows potential relevance to the field of speech impairments considering the high influence that perception deficits exert on production deficits and treatment outcomes in children & adolescents, but also given the absence of clear norms to evaluate children with RSSD. The authors evidently put great effort in the online data recording which was carried out during the COVID-19 pandemic as i) they managed to record data from children and adolescents, ii) they provided the same headphones to all participants iii) they supervised all data recording sessions via zoom calls.

Before recommending for publication, there are some concerns described below, which authors should address. In particular, the main claim of this work is that data recorded online are no less valid than data recorded in person but it is not clear how the results would precisely support such claim. Secondly, authors indicate several limitations of the study in the discussion section which need to be addressed more clearly by providing references and/or additional analyses.

**Major Issues**

1) Authors declare that the aim of the study is to test whether measurements of speech perception for typically developing children performed online are comparable to the ones recorded in a laboratory setting multiple times (lines 123,199). However, in the Limitations section they report that “*The present study was not designed as a prospective comparison of online and in-person settings.*” (line 451-452). It would be appropriate to be clear about the initial aim of the study as soon as possible to help readers in forming a clear picture as well as in understanding how the authors dealt with the continuation of the study despite the multiple problems caused by the COVID-19 pandemic.
2) Line 221: Is parent report a reliable method to check for the presence of developmental disabilities? Can author provide references to support its validity? Are there similar works in which the validity of parent report is compared to the validity of standardized tests or clinical evaluations?
3) Materials and Methods: Details about the psychometric fitting results are missing. Authors should provide the exact fitting formula with all customized parameters (if any, e.g., lapses), plots of the average fitted curves and an appropriate goodness of fit measure. The absence of these details reduces the reproducibility of the study and prevents readers from understanding how well the model fitted to data.
4) Materials and Methods: Is visual inspection a reliable method to determine whether data are normally distributed or not? Author might provide appropriate tests to justify their point. Furthermore, while non-parametric tests are used when comparing means across the online and in-person modalities, linear models are used to evaluate possible effects of different predictors on the dependent variable. A key assumption to implement linear models is normality of model’s residuals. Authors should provide evidence about the normality of residuals to justify the use of linear models. If such condition is not met, they should implement the appropriate analysis method.
5) Discussion: Importantly, author claim that data collected online are no-less valid than data collected in person (lines 541, 542). They back-up this claim by mentioning that identification boundary and category goodness are correlated in the online study but not the in person one (lines 542-545). It is unclear how this finding would justify their claim nor if this specific pattern in the correlation analysis is the only indicator of the validity of data collected online. Relatedly, it is unclear what do the authors exactly mean with the term “validity”. They also suggest that this correlational pattern might be due to different numbers of participants reaching ceiling levels between online and in-person modality. Authors should better clarify the link between results and their interpretations in the discussion section or provide additional analyses/metrics to support their claim (e.g., plotting and/or repeating the analyses by excluding participants showing ceiling performance might be a starting point). This appears as being the central point of the paper, therefore it should be centrally and clearly addressed in the discussion section.
6) Discussion: Results of linear models showed different unexpected patterns concerning several interaction effects of the predictors “Sex”, “Age” and “Modality” which authors mainly attribute to demographic differences in the characteristics of the participants enrolled in the online and the in-person study. It is completely understandable that such differences were not in complete control of researchers considering the COVID-19 pandemic. However, even if statistically significant differences are present in terms of sex and age distributions between online and in-person modalities, considering the sampling bias as the cause of the found interaction effect might be speculative. Authors should attempt at backing-up their claims by providing appropriate evidence from the related literature and/or develop additional analyses to explicitly test if the sampling bias is the source of the unexpected patterns. If possible, authors might try to repeat the analyses by randomly subsampling the in-person database to match the characteristics of the online group in terms of sex and age and verify whether the interaction effects hold. Possible sex differences in speech perception are indeed understudied, thus providing stronger evidence about such patterns might be of relevance to the field.

**Minor Issues**

7) Lines 42-44: The syntax of the sentence is a bit unclear, please rephrase to clarify. Additionally, authors should clarify the meaning of “speech deviation” to facilitate the understanding of non-expert readers.
8) Line 57: Authors should provide an exemplary English word embedding the /ɹ/ sound as they do later in the manuscript.
9) Lines 61-62: Please clarify the meaning of “*the goals driving speech are dynamic regions in auditory-acoustic and somatosensory space”.*
10) Line 66: Please provide a definition for “auditory and somatosensory targets” to help non-expert readers.
11) Line 139: Typo, please correct ``noisier" with “noisier”.
12) Line 212: Please provide a reference or an additional explanation backing up the assumption that a stronger correlation between two measures of the same construct in one modality is an index of the robustness of the assessment in that modality.
13) Line 290: Please fix the citation style of (Ortiz, 2018).
14) Line 314-315: Typo, please correct ``choose whether the /ɹ/ sound is right or wrong" with “choose whether the /ɹ/ sound is right or wrong”.
15) Line 332-334: To facilitate the understanding of the rationale of statistical analyses please do not refer to the “first” and “second” research aim but explicitly report again the aims in words.
16) Line 468: Please be more specific about the result instead of using “higher performance”.
17) Results: While authors provide numerical values of beta coefficients when reporting the results of analyses on the linear models (which are usually self-explanatory), also reporting Mean and Std.Dev/Std.Err. (or other appropriate summary descriptive statistics of choice) of the dependent variables might help readers into mentally representing differences between two/more factor levels.
18) Materials and Methods: In the instruction of the category judgement task authors report that participants saw the “choose whether the /ɹ/ sound is right or wrong” sentence on the screen. Were participants already familiar with the meaning of the “/ɹ/” grapheme or were they given additional explanation about IPA notation? Providing details on instructions, especially in these kinds of tasks developed for children and adolescents might help other researchers in developing efficient instructions
19) Materials and Methods: Were parents present during the assessments in online/in-person modality? Please specify.
20) Factor naming is inconsistent throughout the manuscript. Authors sometime use the term “modality” to refer to the modality in which the experiment was carried out (in-person or online), but some other times they use “condition” (line 373, 387,498) or “group” (line 420, 465). Please use consistent factor and levels naming.
21) Figures: Authors provided density plots to represent the found effects in their statistical analyses but they might not be the most appropriate representations, especially when interaction effects are present. The navigation of the results might be facilitated by adding additional figures employing e.g., boxplots/violin plots for categorical variables (e.g., effect of sex) and e.g., points and lines plots when representing the effect of a continuous variable (e.g., age).
